# No differences in therapeutic efficacy while sparing healthy tissue for orthotopic glioblastoma patient-derived xenografts in context of proton FLASH

**DOI:** 10.1016/j.ctro.2025.101050

**Published:** 2025-09-19

**Authors:** Taylor L. Schanel, Manoj Kumar, Lauren C. Nassour-Caswell, Sreelakshmi Cherakara, Rhea Pandit, Andee M. Beierle, Joshua C. Anderson, Patrica H. Hicks, Rex Cardan, Anita B. Hjelmeland, Christopher D. Willey

**Affiliations:** a Department of Radiation Oncology, Heersink School of Medicine, University of Alabama at Birmingham, Birmingham, AL 35233, USA; b Department of Cellular, Developmental, and Integrative Biology, University of Alabama at Birmingham, Birmingham, AL 35233, USA

**Keywords:** FLASH-RT, Ultra-high dose rate radiation, Proton beam therapy, Conventional RT, Radiotherapy, Glioblastoma, Patient-derived xenografts

## Abstract

Ultra-high dose rate (FLASH-RT) and conventional proton beam radiotherapy to intracranial glioblastoma PDX were compared for tumor control and normal tissue toxicity via DNA and RNA damage response markers. Both conventional and FLASH radiotherapy yielded similar survival benefits; however, conventional radiotherapy resulted in greater normal tissue DNA and RNA damage.

## Highlights

• Glioblastoma (GBM) brain tumor initiating cells (BTIC) were tested for FLASH-RT effect in patient-derived xenograft (PDX).
• Identification of equipotency of FLASH-RT and CONV-RT protons in BTIC-compliant orthotopic GBM PDX model.
• Identification of normal brain tissue sparing using FLASH-RT compared to CONV-RT.

## Introduction

Patients diagnosed with the primary malignant brain tumor, glioblastoma (GBM), receive standard of care therapy consisting of maximal safe resection followed by fractionated radiation and concurrent temozolomide (TMZ) chemotherapy, and maintenance TMZ therapy[1]. X-ray based radiotherapy (RT) is one of the few treatment options that has markedly enhanced survival, doubling survival time in patients who receive irradiation compared to surgery alone[[2], [3], [4]]. However, radiation dose is limited by the harmful effects on non-cancerous tissue such as astrocytes, neurons, and microglial[5] leading to a negative impact on cognitive function.

Ultra-high dose rate (e.g., >40 Gy/s) “FLASH” RT is a promising approach to reduce normal tissue toxicity when compared to equal doses of conventional dose rate (e.g., <0.1 Gy/s) RT[6,7]. Over the past decade, multiple pre-clinical studies have demonstrated the “FLASH effect,” where the delivery of high-dose rate radiation preserves normal tissue without compromising tumor control[[8], [9], [10], [11], [12]]. Several mechanisms have been proposed including transient oxygen depletion, free-radical recombination, reduced reactive oxygen species, reduced immune cell disruption, and stem cell preservation [13]. While several elegant neurobehavioral studies have confirmed reduced normal brain injury with FLASH-RT [[14], [15], [16], [17]], the GBM models published, thus far, utilize immortalized cell culture models that notably lack brain tumor initiating cells (BTIC), cancer stem-like cells. As such, there is concern that FLASH-RT may also preserve BTICs thereby reducing the therapeutic index for FLASH-RT. Another scientific gap is the FLASH-RT delivery method. Most studies using FLASH-RT have relied on electron beam delivery approaches which have some practical limitations, particularly for deep seated tumors, that could be overcome by proton beam therapy. So, while electron FLASH-RT is more widely available for preclinical use, the first human clinical trial for FLASH-RT (FAST-001) utilized transmission beam proton delivery. To address these concerns, we tested an intracranial GBM patient derived xenograft (PDX) model that maintains BTIC biology and is known to better represent human disease to assess FLASH-RT versus conventional RT (CONV-RT) using a clinical proton facility (Varian ProBeam).

## Materials and methods

Full materials and methods can be found in the supplementary material.

### Tumor model

GBM PDX model (XD456) was acquired through the University of Alabama at Birmingham Brain Tumor Animal Model Core originally provided as a gift from Dr. Darrel Bigner at Duke University. XD456 cells were infected with serum-free luciferase lentivirus vector pGreenFire1-CMV (System Biosciences, Catalog #TR011PA-1) prepared using CSC293T cells as previously described [[18], [19], [20]] in the Hjelmeland Laboratory at UAB. For in vitro PDX propagation, XD456 luciferase^+^ cells were cultured in serum free medium as before [21].

### Animal experiments

Twenty-eight four-week-old female athymic nude mice (nu/nu) were purchased from Charles River Laboratories. Procedures were performed under IACUC-21935 approval. Orthotopic injections were performed as before [22,23]. Briefly, mice were anesthetized with isoflurane (VETone Fluriso, Catalog #NDC 13985–528-60) and XD456 luciferase^+^ cells (2 x 10^4^) suspended in 10 μL DMEM/F12 were injected in the right frontal lobe to a depth of 2 mm. Animals were monitored for 30 min post-injection. Survival was monitored through daily checks for physical health deformities (weight loss, hunch back, and decreased mobility). Tumor burden was monitored with bioluminescent imaging. At study termination, animals were euthanized by isoflurane inhalation and cervical dislocation.

### Irradiation device and treatment administration

To address limitations of the Varian ProBeam’s research transmission chamber in accurately assigning clinically calibrated monitor units (MUs) at FLASH dose rates, a calibration procedure was performed before each irradiation session. A relationship between the transmission ion chamber response and the physical dose output was established for a range of output currents (10nA to 90nA). This was achieved by positioning a PPC05 parallel-plate ionization chamber (IBA, Louvain-La-Neuve, Belgium) at isocenter under standard clinical calibration conditions. A FLASH scaling factor was then determined by delivering a 10 cm x 10 cm uniform spot field at both clinical and FLASH dose rates. This factor allowed us to correlate requested MUs with delivered MUs for the rates used throughout the experiment. Following calibration, irradiation plans were generated and uploaded to the Varian Racehorse software, a treatment planning and delivery system, in the form of ASCII files. Prior to delivery for each mouse plan, a single spot at clinical dose rates was delivered, followed by a single spot delivered at FLASH dose rates using the equivalent MU from the prior established relationship to verify equivalent dose to the ion chamber. Since the dose varies greatly across the spot profile, the central axis (peak) dose was chosen as the point to map dose to MU. All reported doses therefore are at the center of the spot and falloff on a semi-Guassian with a FWHM of 7 mm. During delivery, the ion chamber was placed behind the mouse on the central axis and dose was recorded for delivery. The time of delivery for each spot was provided by Varian internal log file analysis software. Animals were separated into two cohorts (cohort 1, n = 15 and cohort 2, n = 13) based on tumor volume from BLI and were anesthetized with 100 mg/kg ketamine and 10 mg/kg xylazine. Animals were then either administered CONV-RT dose rate or FLASH-RT dose rate for a single total dose administration of 10 Gy. Animals were monitored for 30 min post-injection. Cohort 2 was treated one week after cohort 1.

### Bioluminescence imaging (BLI)

Tumor burden was monitored by BLI 10 days post orthotopic injection, and every three days post-treatment for day 1, 4, 7, 10, 13, 16, 19, 21, 24, and 27. Mice were imaged using IVIS Lumina III in UAB Small Animal Imaging Facility.

### Immunocytochemistry

Primary antibodies γH2AX (Abcam, Catalog #ab11174) concentration of 1:500 and DNA/RNA Damage (Abcam, Catalog #ab62623) concentration 1:500 was incubated overnight 4 °C. Secondary antibodies Donkey Anti-Rabbit Alexa Fluor 594 (Abcam, Catalog #ab150076) concentration 1:1000 and Donkey Anti-Mouse 568 (Invitrogen, Catalog #A10037) concentration 1:1000 were incubated for 30 min at room temperate. HOECHST was incubated for 10 min at room temperature with a concentration of 10 mg/mL dilution 1:1000 (ThermoFisher, Catalog #33258). Slides were mounted with Immu-Mount (Epredia, Catalog #9990402).

### Statistical analysis

Animal experiments were conducted in two cohorts (n = 15 and n = 13). Data analysis was performed using Prism 10.4.1 (GraphPad) and the relevant statistical tests are indicated in the respective figure legends. For Kaplan-Meier survival curves, a log-rank (Mantel-Cox) was performed with reported p values.

## Results

### FLASH-RT is isoeffective to CONV-RT in GBM PDX Model

**Fig. 1:**
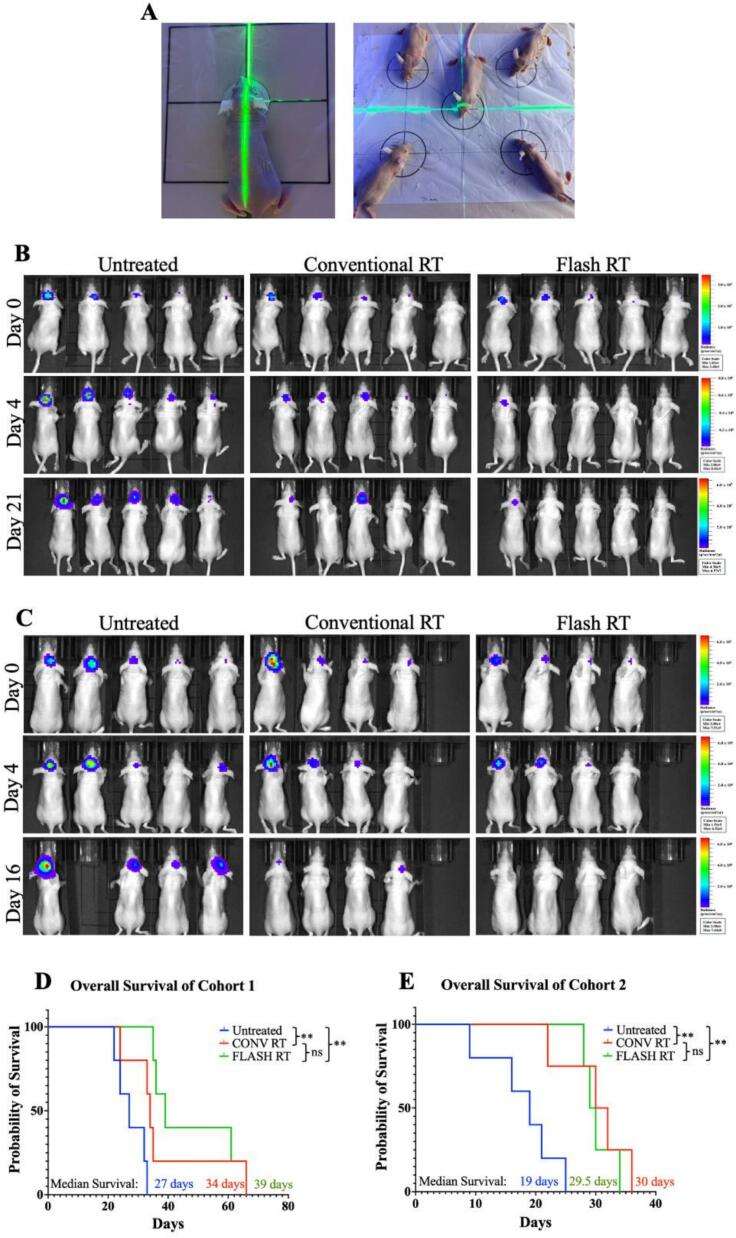
**CONV and FLASH RT increases survival and decreases tumor burden compared to no treatment with no significant difference in survival between CONV and FLASH RT.** (A) Representative images displaying the layout for RT administration targeting the right frontal lobe region using a pre-designed beam trajectory. (B) Bioluminescent images representative of cohort 1 mice tumor-burden tracking over 21 days post RT. (C) Bioluminescent images representative of cohort 2 mice tumor-burden tracking over 16 days post RT. Kaplan-Meier survival curve of (D) cohort 1 (n = 15, n = 5 untreated, n = 5 CONV RT, n = 5 FLASH RT) and (E) cohort 2 (n = 13, n = 5 untreated, n = 4 CONV RT, FLASH RT m = 4). The log-rank (Mantel-Cox) test showed statistically significant survival difference in both cohorts between untreated groups and the CONV RT / FLASH RT groups. Cohort 1 (** p = 0.0028) and cohort 2 (**p = 0.0018). The log rank test indicated no statically significant survival difference in both cohorts between CONV RT and FLASH RT. The median survival time in days is shown for each cohort of mice. Untreated (blue line), CONV RT (red line) and FLASH RT (green line).

To assess the difference between CONV and FLASH dose rate effects with proton beams, tumor-bearing mice were irradiated with either pulsed delivery over 5 min (CONV-RT) or FLASH-RT for a single administration of 10 Gy. Both CONV and FLASH groups received a single treatment session utilizing a pre-designed beam trajectory to ensure consistent RT delivery across conditions (Fig. 1A). BLI was used to assess tumor burden in luciferase^+^ XD456 tumor-bearing mice over 21 days for cohort 1 and 16 days for cohort 2 (Fig. 1, Fig. 1, and Supplemental Fig. 1). Bioluminescent signal was detectable in mice 10 days post orthotopic injection, indicated as Day 0. Treatment was administered 24hrs after imaging of Day 0. The assessment of tumor burden post-RT on Day 4 is presented in the middle panels (Fig. 1, Fig. 1). Control mice had a progressive increase in bioluminescent signal over time, indicating tumor progression (Fig. 1, Fig. 1). The signal intensity in the CONV-RT mice appeared slightly reduced compared to untreated (Fig. 1, Fig. 1). In contrast, the FLASH RT group exhibited decreased bioluminescence signal at Day 21, suggesting reduced tumor burden (Fig. 1, Fig. 1).

Overall survival was assessed in two independent cohorts following untreated, CONV-RT, or FLASH-RT treated animals (Fig. 1, Fig. 1). In cohort 1, mice receiving FLASH-RT showed no difference in survival compared to CONV RT, while both RT groups had significantly increased survival relative to untreated controls (p = 0.0028). Median survival times were 27 days for untreated, 34 days for CONV RT, and 39 days for FLASH RT, indicating at least equal efficacy of FLASH RT in this cohort. We saw a similar response in cohort 2 with both CONV and FLASH RT groups having significantly increased survival compared to untreated controls (p = 0.0018), but no significant difference between FLASH and CONV-RT.

### Decreased Normal Tissue DNA Damage with FLASH-RT

**Fig. 2:**
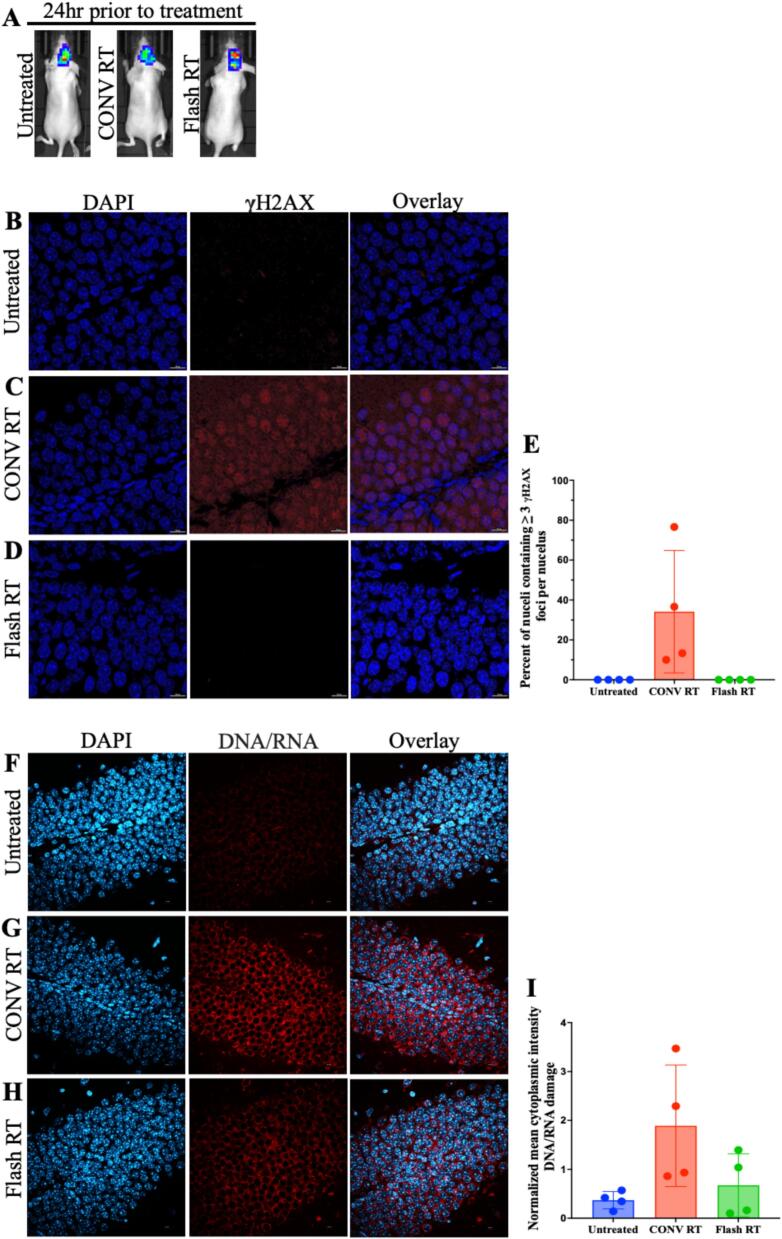
**FLASH RT demonstrates decreased cellular damage markers compared to CONV RT.** (A) Representative bioluminescent images of tumor-bearing mice whose brains were collected 24 hrs post whole-brain RT treatment. Immunofluorescent confocal imaging of the hippocampal dentate gyrus region of paraffin-embedded athymic nude mice brains treated with either CONV RT (C, G) or FLASH RT (D, H). Untreated mice (B, F) received no radiation treatment. Sections (B, C, D) were stained for DNA damage marker **γ**H2AX antibody (red) and nuclear stain with DAPI (blue). Nuclei with ≥ 3 foci out of thirty total cells were counted as positive for **γ**H2AX foci staining (E). Magnification 60x. Scale bar is 10 μm. Nuclei were quantified over four acquired images in FIJI (E). Sections (F, G, H) were stained for DNA/RNA damage markers 8-OHdG, 8-OHG, and 8-oxoG (red) and nuclear stain with DAPI (blue). Mean total fluorescence intensity of the red channel for DNA/RNA damage markers is indicated with quantification per cell divided by the total number of cells over four acquired images in FIJI (I). Magnification 40x. Scale bar is 10 μm.

Tumor-bearing mice were treated with single 10 Gy dose at either CONV-RT or FLASH-RT dose rate. Tumor burden was measured twenty-four hours prior to treatment (Fig. 2A). Whole brain excision was performed twenty-four hours post-treatment for immunocytochemistry analysis. Gamma H2AX (γH2AX), a marker for DNA double strand breaks, important for its role in initiating and facilitating DNA repair was probed to assess DNA damage following CONV and FLASH-RT (Fig. 2, Fig. 2, Fig. 2 and Supplemental Fig. 2) in the irradiated dentate gyrus. Untreated and FLASH-RT samples demonstrate minimal γH2AX foci, indicating little DNA damage (2B, 2D, 2E). However, the CONV-RT sample demonstrates an increased of γH2AX foci positive cells (≥3 foci/nucleus) indicating increased DNA damage 24 hrs post RT in comparison to untreated and FLASH-RT (2C, 2E). Further we aimed to assess nucleic acid damage within the cytoplasm, indicated by oxidative stress markers 8-hydroxy-2′-deoxyguanosine, 8-hydroxyguanosine, and 8-hydroxyguanine which have been shown to be induced by RT[24]. Untreated sample showed decreased detection of DNA/RNA damage marker compared to CONV and FLASH-RT (Fig. 2, Fig. 2 and Supplemental Fig. 3), but we found no statistically significant difference in DNA/RNA damage between CONV RT and Flash RT groups (Fig. 2, Fig. 2).

## Discussion

Despite promising preclinical and veterinary studies suggesting FLASH-RT could be a potential strategy for treating a variety of tumors including GBM, fundamental questions remain unanswered which may hinder the development and utilization of FLASH-RT. A problem of high importance is the considerable lack of preclinical studies using advanced patient-derived models of cancer. Indeed, there is a concern in the field that if FLASH preferentially protects normal tissue stem cells, then cancer stem cells, which are poorly represented in traditional immortalized cell lines, would be protected as well. Our study represents one of the few investigations to utilize PDX rather than immortalized cell lines to evaluate the FLASH effect [25], and the first to do so in GBM. We determined that single fraction FLASH-RT was equally effective to CONV-RT in terms of intracranial survival benefit in a BTIC-compliant GBM preclinical model while also confirming normal tissue sparing as evidenced by reduced markers of DNA damage at 24 hrs. These encouraging results suggest that proton FLASH-RT can spare normal brain tissue from radiation injury while providing similar tumor control rates in human GBM patient-derived models further supporting clinical development of FLASH-RT in patients. Moreover, by using a clinical proton machine, our approach enhances the clinical relevance of our findings and suggests a therapeutic potential of FLASH RT.

Limitations of our approach include using the transmission portion of the proton beam rather than the Bragg peak and the use of pulsed-dose proton therapy to achieve “conventional” dose rate. The physical advantage of proton radiation relies on the spread-out Bragg peak used in clinical scenarios though achieving this for FLASH-RT is only beginning to emerge [[26], [27], [28], [29]]. Future work utilizing Bragg Peak delivery of FLASH-RT might further improve therapeutic index. CONV-RT was achieved through pulsing of lower current (10nA) proton beam output with individual pulses on the order of ∼ 10 Gy/s but split over several minutes to achieve a mean dose rate in the clinical range. Ideally, the CONV-RT would be delivered in a continuous fashion, but we chose the split pulse method which allowed for more consistent dosimetry across groups and better controlled mouse handling due to significant time to change from FLASH-mode to Clinical-mode with sufficient calibration. Despite these shortcomings, our work still demonstrated the FLASH effect in the preferred preclinical model for GBM − intracranial PDX.

Our findings align with those of Montay-Gruell et al. [17], who demonstrated similar FLASH-RT outcomes in non-PDX models, which generally contain few BTICs. Given that BTICs are key drivers of radioresistance and recurrence, understanding their response to ultra-high dose rate irradiation is critical. Although we did not directly quantify BTIC populations in this study, BTICs may respond differently to FLASH than non-BTIC tumor cells, potentially affecting DNA repair, stemness, cell cycle regulation, hypoxic niche interactions, and immune modulation. Future studies should prioritize models with defined BTIC content, including both PDX and non-PDX systems, to directly assess BTIC-specific responses and clarify whether the FLASH effect extends to cancer stem cells, informing strategies to prevent tumor recurrence.

In summary, our data support the dual benefit of FLASH-RT in both reducing tumor burden and minimizing normal tissue toxicity. The observed reduction in double-strand break markers in FLASH-RT treated animals provides mechanistic insight into the protective effects on healthy brain tissue.

## Declaration of competing interest

The authors declare the following financial interests/personal relationships which may be considered as potential competing interests: This work was supported by the Varian FLASH Forward Consortium (to C.D.W. and A.B.H).
