# Supplementary material for: No differences in therapeutic efficacy while sparing healthy tissue for orthotopic glioblastoma patient-derived xenografts in context of proton FLASH
Source: Clin Transl Radiat Oncol. 2025 Sep 19;56:101050. doi: 10.1016/j.ctro.2025.101050 (PMC12489934; doi:10.1016/j.ctro.2025.101050)
Supplement: Supplementary Data 1 [file mmc1.docx]

Supplemental Methods:

Tumor model

Serum free medium consists of DMEM/F12 50/50 (Corning, Cat. #10–090-CV) supplemented with B27 (Gibco, Cat. #17504044), 1 % Sodium Pyruvate (Corning, Cat. #5000CI), bEGF (20 ng/ml) (Gibco, Cat. #PHG0311), bFGF (20 ng/ml) (Gibco, Cat. #PHG0261), and 1 % penicillin/streptomycin (Corning, Cat. #30–001-CI).

Animal Experiments

Mice were housed under a 12 hour light:dark cycle at 20-23°C in an explicit pathogen-free facility and supplied with enrichment, standard chow food and water ad libitum.

Bioluminescence imaging

Mice were injected with 100uL of 2.5mg D-luciferin/saline solution (GoldBio, Catalog #LUCK-1G) into the peritoneal cavity. After a 10 min delay for systemic circulation. Animals were imaged in dorsal prone position, with five mice imaged per sequence using a 1 sec exposure and at 24 cm camera distance.

Immunocytochemistry

Mice were euthanized by isoflurane inhalation and cervical dislocation. Whole brains were dissected and fixed in 1:10 diluted buffered histology grade formalin (Fisher Scientific, Catalog #305-510) for 24 hours. Fixed tissue was stored in 70% histology-grade ethanol (Decom Labs Catalog #04-355-223) at room temperature until tissue processing. Tissue samples were trimmed, paraffin-embedded, and sectioned at the UAB Comparative Pathology Laboratory. Tissue was sectioned via microtome at 5 µm sections. Every other section was and placed onto charged microscopes slides. Slides were stored at 4°C until staining. Heat-induced antigen retrieval was performed for 30 minutes with Antigen Unmasking Solution, Citric Acid Based (Vector Laboratories, Catalog #ZL0613) at 100°C. Tissue was permeabilized with a solution of 0.2% Tirton X (Fisher Scientific, Catalog #BP151-500) in PBS (Corning, Catalog #21-040-CM) for 10 minutes at room temperature. Blocking solution of 0.1% Triton X, 1% BSA (Sigma-Aldrich, Cat. #0000291524), 1% Donkey Serum (Sigma-Aldrich, Catalog #D9663-10ML), and sodium azide (Fisher Scientific, Catalog #AAJ2161036), in PBS incubated for 1 hour at room temperature.

Confocal microscopy image acquisition

γH2A samples were imaged using a Nikon Ti2 inverted fluorescence microscope equipped with a tandem galvano scanner and a Nikon A1R-HD25 resonance scanner. Images were captured at 1024x1024 or 2048x2048 Nyquist-sampled pixel resolutions using an Apo 60x/1.4 NA Gamma oil DIC WD 140 objectives. Fluorophores were excited with the following lasers: γH2A with a 594 nm laser and DAPI with a 405 nm laser. Imaging was performed using NIS software. DNA/RNA damage samples were imaged using a Nikon Spinning-disk confocal microscope with Yokogawa X1 disk, using Hamamatsu flash4 sCMOS camera. Images were captured with a 605/52 filter using a Plan Fluor 40x Oil DIC H N2; NA=1.30, WD=240 µm. Fluorophores were excited with the following lasers: DNA/RNA damage with a 568 nm laser and DAPI with a 405 nm laser.

Percent of nuclei containing > 3 foci per nucleus analysis

// Fiji Macro: Open file, split channels, set γH2AX contrast

// --- Step 1: Open file ---

open("file name");

// --- Step 2: Split channels ---

run("Split Channels");

// --- Step 3: Rename channels (assumes C1 = DAPI, C2 = γH2AX) ---

selectWindow("C1-60x_ConIR_yh2a_1d_hippocampus.nd2");

rename("DAPI");

selectWindow("C2-60x_ConIR_yh2a_1d_hippocampus.nd2");

rename("gH2AX");

// --- Step 4: Set brightness/contrast for γH2AX ---

selectWindow("gH2AX");

setMinAndMax(1187, 1969);

// --- Step 5: Find Maxima on γH2AX channel ---

run("Find Maxima...", "noise=1100 output=[Point Selection]");

// --- Step 6: Create merged composite (DAPI + gH2AX) ---

selectWindow("DAPI");

run("Merge Channels...", "c1=DAPI c2=gH2AX create");

rename("Merged");

// --- Step 7: Transfer maxima points onto merged image ---

selectWindow("Merged");

roiManager("reset");

roiManager("Add"); // add the point selection to ROI Manager

roiManager("Show All"); // display points overlayed

Cytoplasmic DNA/RNA damage total mean fluorescent intensity per cell normalized to the total number of cells analysis

// FIJI Macro: Measure Cytoplasmic Intensity Per Cell in Red Channel (TIFF)

// Adjusts Brightness/Contrast (min=46, max=255) before measuring

// === Step 1: Open Red Channel TIFF ===

open("Sample 9_DG2_Red.tif"); // <-- Change to your actual TIFF file name

// === Step 2: Set Brightness/Contrast for Red Channel ===

setMinAndMax(46, 255);

run("Apply LUT");

// === Step 3: Convert to 8-bit Grayscale ===

run("8-bit");

// === Step 4: Segment Cells ===

// (Basic segmentation - you may want to refine for your data)

run("Gaussian Blur...", "sigma=2");

setAutoThreshold("0,24"); // Adjust threshold range if needed

run("Convert to Mask");

run("Watershed"); // Separate touching cells

run("Analyze Particles...", "size=50-Infinity show=Nothing add"); // Adjust size range

// === Step 5: Measure Cytoplasmic Intensity ===

run("Set Measurements...", "mean area integrated redirect=None decimal=3");

run("Measure");

// === Optional: Close intermediate images ===

// close("*");
