# Supplementary material for: No differences in therapeutic efficacy while sparing healthy tissue for orthotopic glioblastoma patient-derived xenografts in context of proton FLASH
Source: Clin Transl Radiat Oncol. 2025 Sep 19;56:101050. doi: 10.1016/j.ctro.2025.101050 (PMC12489934; doi:10.1016/j.ctro.2025.101050)
Supplement: Supplementary Data 2 [file mmc2.pdf]

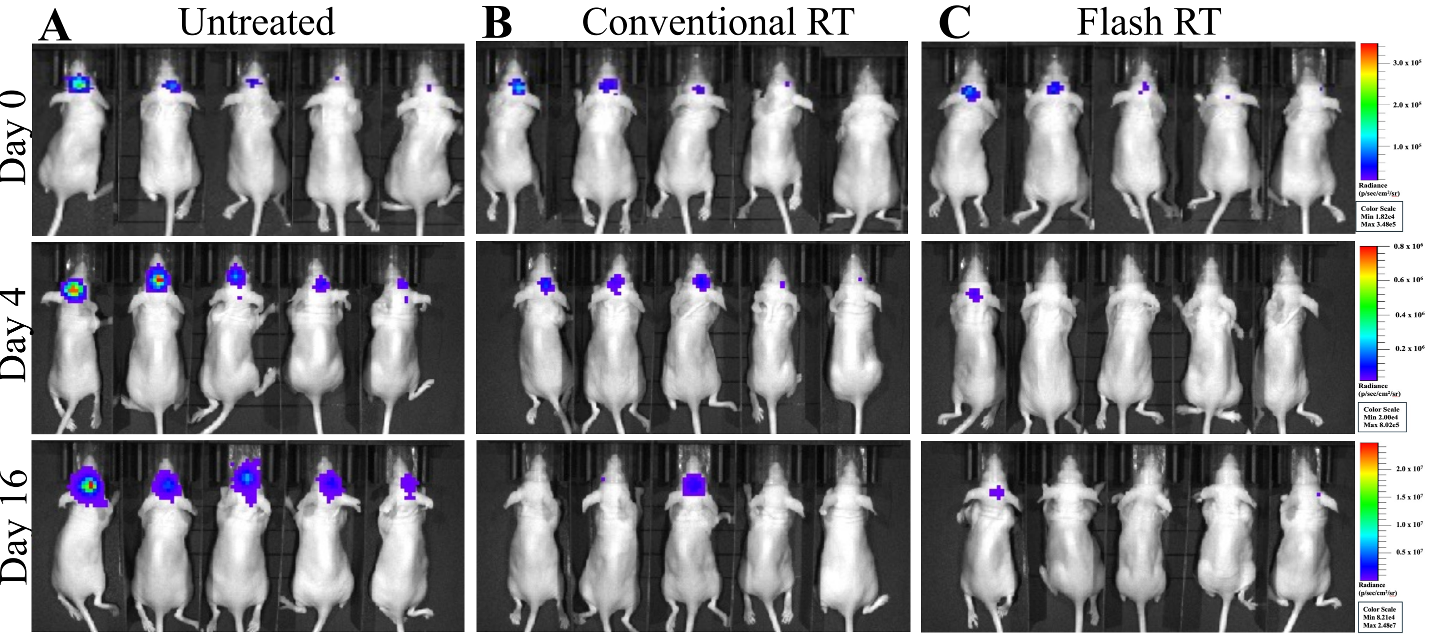

Supplemental Figure 1. Tumor burden decreases over 16 days in CONV RT and FLASH RT treatment mice compared to untreated control. Bioluminescent images representative of cohort 1 mice tumor-burden tracking over 16 days post RT.

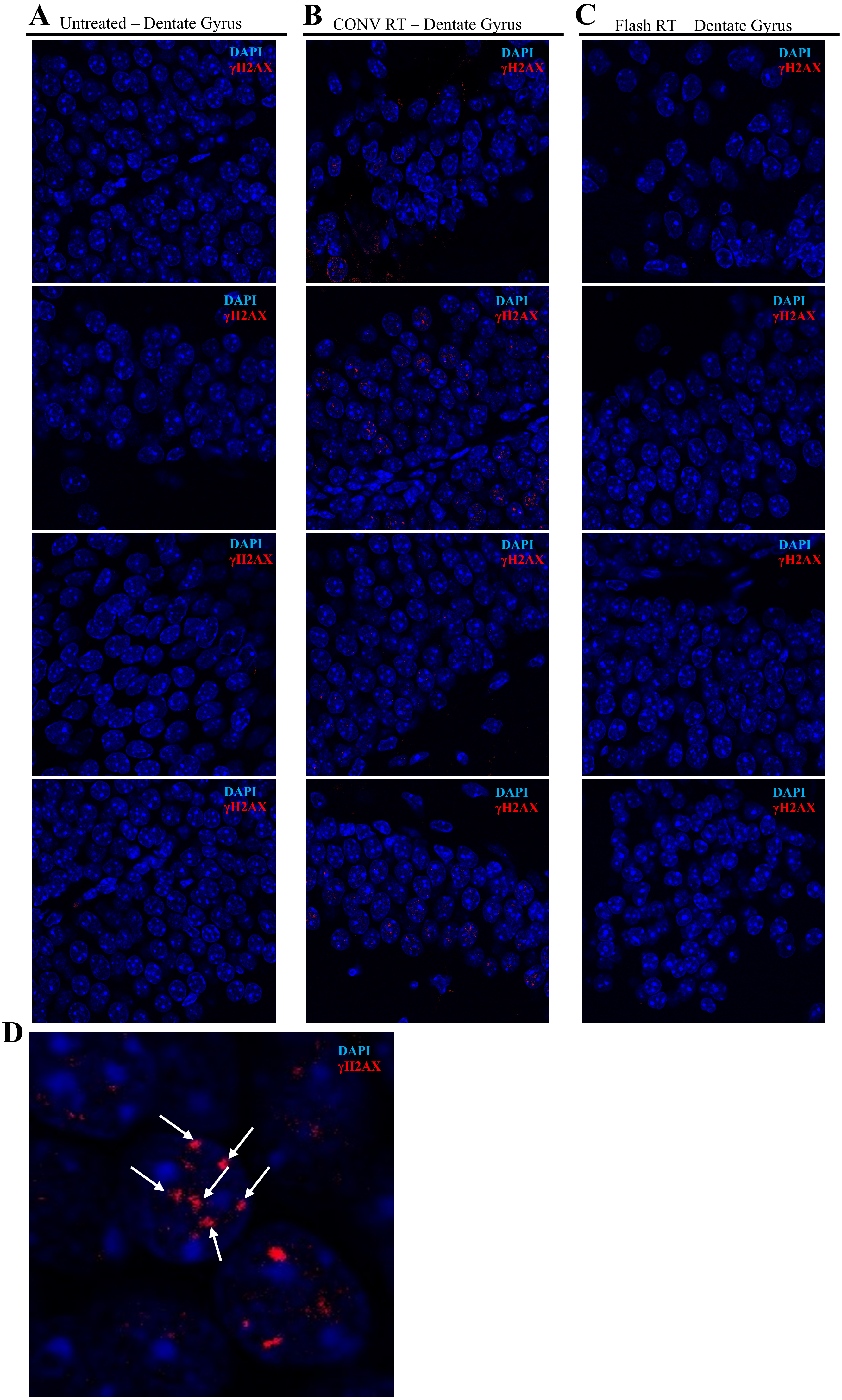

Supplemental Figure 2. FLASH RT demonstrates decreased γH2AX foci compared to CONV RT. Immunofluorescent confocal imaging of the hippocampal dentate gyrus region of paraffin-embedded athymic nude mice brains from animals treated with either CONV RT (B) or FLASH RT (C). Untreated mice (A) received no radiation treatment. Sections were stained for DNA damage marker γH2AX antibody (red) and nuclear stain with DAPI (blue). (A, B, C) Merged DAPI and γH2AX channel images demonstrating the overlay of γH2AX foci location (D). Representative image of defined positive γH2AX foci staining in a single nucleus (white arrows). Four technical replicates were taken per group. Magnification 60x. Scale bar is 10µm.

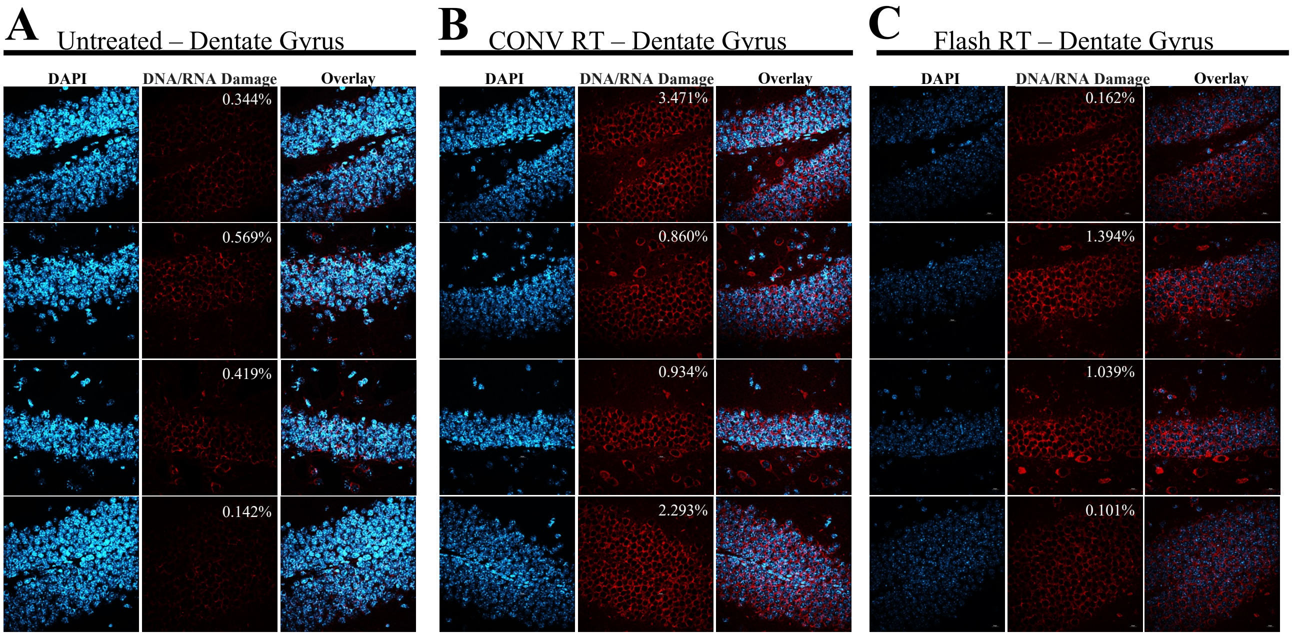

Supplemental Figure 3. Cellular damage markers comparing untreated, CONV RT, and Flash RT. Immunofluorescent confocal imaging of the hippocampal dentate gyrus region of paraffin-embedded athymic nude mice brains from animals treated with either CONV RT (B) or FLASH RT (C). Untreated mice (A) received no radiation treatment. Sections were stained for DNA/RNA damage makers 8-OHdG, 8-OHG, and 8-oxoG (red) and nuclear stain with DAPI (blue). Magnification 40x. Scale bar is 10µm. Mean total fluorescence intensity of the red channel for DNA/RNA damage markers is indicated with quantification per cell divided by the total number of cells over four acquired images in FIJI indicated as a percent value (white).
